# Supplementary material for: HIVprotI: an integrated web based platform for prediction and design of HIV proteins inhibitors
Source: J Cheminform. 2018 Mar 9;10:12. doi: 10.1186/s13321-018-0266-y (PMC5845081; doi:10.1186/s13321-018-0266-y)
Supplement: Supplementary file 2 — Additional file 2. Source code of HIVProtI web server. [file 13321_2018_266_MOESM2_ESM.zip › HIVprotI_Source-code/hivprotI/weka-3-6-6/doc/weka/classifiers/lazy/kstar/KStarNumericAttribute.html]

KStarNumericAttribute


---


|  |  |  |
| --- | --- | --- |
| **Overview** \| **Package** \| **Class** \| **Tree** \| **Deprecated** \| **Index** \| **Help** \| **Weka's home** | |  |
| **PREV CLASS**   **NEXT CLASS** | **FRAMES**    **NO FRAMES**     **All Classes** |  |
| SUMMARY: NESTED \| FIELD \| CONSTR \| METHOD | DETAIL: FIELD \| CONSTR \| METHOD |  |


---


## weka.classifiers.lazy.kstar Class KStarNumericAttribute

```
java.lang.Object
  weka.classifiers.lazy.kstar.KStarNumericAttribute
```

**All Implemented Interfaces:**: KStarConstants, RevisionHandler

---

``` public class KStarNumericAttribute extends java.lang.Object implements KStarConstants, RevisionHandler ```

A custom class which provides the environment for computing the
transformation probability of a specified test instance numeric
attribute to a specified train instance numeric attribute.

**Version:**
:   $Revision 1.0 $

**Author:**
:   Len Trigg (len@reeltwo.com), Abdelaziz Mahoui (am14@cs.waikato.ac.nz)

---

**Field Summary**

**Fields inherited from interface weka.classifiers.lazy.kstar.KStarConstants**

`B_ENTROPY, B_SPHERE, EPSILON, FLOOR, FLOOR1, INITIAL_STEP, LOG2, M_AVERAGE, M_DELETE, M_MAXDIFF, M_NORMAL, NUM_RAND_COLS, OFF, ON, ROOT_FINDER_ACCURACY, ROOT_FINDER_MAX_ITER`


**Constructor Summary**

`KStarNumericAttribute(Instance test,
Instance train,
int attrIndex,
Instances trainSet,
int[][] randClassCols,
KStarCache cache)`
  
          Constructor


| **Method Summary** | |
| --- | --- |
| `java.lang.String` | `getRevision()`             Returns the revision string. |
| `void` | `setBlendFactor(int factor)`             Set the blending factor |
| `void` | `setBlendMethod(int method)`             Set the blending method |
| `void` | `setMissingMode(int mode)`             Set the missing value mode. |
| `void` | `setOptions(int missingmode, int blendmethod, int blendfactor)`             Set options. |
| `double` | `transProb()`             Calculates the transformation probability of the attribute indexed "m\_AttrIndex" in test instance "m\_Test" to the same attribute in the train instance "m\_Train". |

**Methods inherited from class java.lang.Object**

`equals, getClass, hashCode, notify, notifyAll, toString, wait, wait, wait`

**Constructor Detail**

### KStarNumericAttribute

```
public KStarNumericAttribute(Instance test,
                             Instance train,
                             int attrIndex,
                             Instances trainSet,
                             int[][] randClassCols,
                             KStarCache cache)
```

:   Constructor


**Method Detail**

### transProb

```
public double transProb()
```

:   Calculates the transformation probability of the attribute indexed
    "m\_AttrIndex" in test instance "m\_Test" to the same attribute in
    the train instance "m\_Train".

    :   **Returns:**: the probability value

---


### setOptions

```
public void setOptions(int missingmode,
                       int blendmethod,
                       int blendfactor)
```

:   Set options.

    :   **Parameters:**: `missingmode` - the missing value treatment to use: `blendmethod` - the blending method to use: `blendfactor` - the level of blending to use

---


### setMissingMode

```
public void setMissingMode(int mode)
```

:   Set the missing value mode.

    :   **Parameters:**: `mode` - the type of missing value treatment to use

---


### setBlendMethod

```
public void setBlendMethod(int method)
```

:   Set the blending method

    :   **Parameters:**: `method` - the blending method to use

---


### setBlendFactor

```
public void setBlendFactor(int factor)
```

:   Set the blending factor

    :   **Parameters:**: `factor` - the level of blending to use

---


### getRevision

```
public java.lang.String getRevision()
```

:   Returns the revision string.

    :   **Specified by:**: `getRevision` in interface `RevisionHandler`
    :   **Returns:**: the revision


---


|  |  |  |
| --- | --- | --- |
| **Overview** \| **Package** \| **Class** \| **Tree** \| **Deprecated** \| **Index** \| **Help** \| **Weka's home** | |  |
| **PREV CLASS**   **NEXT CLASS** | **FRAMES**    **NO FRAMES**     **All Classes** |  |
| SUMMARY: NESTED \| FIELD \| CONSTR \| METHOD | DETAIL: FIELD \| CONSTR \| METHOD |  |


---
